# Non-diabetic metabolic nodular glomerulosclerosis 

**DOI:** 10.5414/CNCS110943

**Published:** 2022-12-02

**Authors:** Catarina Mateus, Eunice Cacheira, Ivo Laranjinha, Jorge Dickson, Augusta Gaspar

**Affiliations:** Nephrology Department, Hospital Santa Cruz, Av. Prof. Dr. Reinaldo dos Santos, Carnaxide, Portugal

**Keywords:** nodular glomerulosclerosis, idiopathic nodular glomerulosclerosis

## Abstract

Nodular glomerulosclerosis is classically associated with diabetes. Nowadays, it is well known that this histologic pattern can be the presentation of different diseases, including dysproteinemias and amyloidosis. Most recently, the previously thought to be idiopathic nodular glomerulosclerosis has been associated with hypertension, smoking, and obesity. We present a clinical case of a non-diabetic 74-year-old man, with hypertension and heavy smoking history, who presented with nephrotic proteinuria and chronic kidney disease. We review the literature and propose a different nomenclature for this pattern of metabolic glomerulopathy.

## Introduction 

Nodular glomerulosclerosis is a histologic pattern historically associated with diabetic nephropathy. It was first described in 1936 by Kimmelstiel and Wilson [1] as intercapillary glomerulosclerosis related to diabetes. As with almost all the histologic patterns, these lesions are not pathognomonic, and the final diagnosis implies the integration of clinical data. 

Nodular glomerulosclerosis has been associated with numerous other conditions, so it requires an extensive laboratory work-up and rigorous histologic evaluation of renal biopsy, including histochemistry, immunofluorescence, and electron microscopy. 

In the past, when no cause was found to explain this diabetic-like nephropathy, it was called idiopathic nodular glomerulosclerosis [2]. In 1999, Herzenberg et al. [2] observed that hypertension and obesity were common among these patients, and in 2002, an association with smoking was suggested for the first time by Markowitz et al. [3]. Also, this type of lesion has been described in patients with impaired fasting glucose or impaired glucose tolerance, and in some cases was described as the initial diabetes manifestation [4]. 

We present a case of non-diabetic metabolic nodular glomerulosclerosis (NDMNG) and a review of the literature. 

## Case report 

A 74-year-old Caucasian man was referred to nephrology consultation for poorly controlled hypertension (imprecise starting date) and kidney dysfunction, with a serum creatinine of 1.57 mg/dL (eGFR 53 mL/min/1.73m^2^). 

Besides hypertension, his cardiovascular history included heart failure with left ventricular hypertrophy with preserved ejection fraction and moderate aortic insufficiency. Of note, there was an ongoing heavy cigarette smoking (60 pack-years). Other relevant data included asymptomatic benign prostatic hyperplasia and nephrolithiasis, with episodes of renal colic in the past. He was under furosemide, perindopril, amlodipine, nebivolol, rilmenidine, simvastatin, and tamsulosin, but no regular NSAIDs use. 

Physical examination revealed blood pressure of 178/57 mmHg and no peripheral edema, a weight of 58 kg, and a body mass index of 21 kg/m^2^. 

Urinalysis showed a protein-to-creatinine ratio of 6,042 mg/g, an albumin-to-creatinine ratio of 4,603 mg/g, and microscopic hematuria. Hypercholesterolemia (total cholesterol 242 mg/dL) and hyperuricemia (serum uric acid 7.1 mg/dL) were present; sedimentation rate was 67 mm/h. Serum albumin was within the normal range. The serum free light chain ratio, total IgG IgA, and IgM were normal, serum protein electrophoresis showed no M spike, and immunofixation was negative. The immunologic study was also negative (complement, rheumatoid factor, ANA’s, anti-dsDNA, ANCA), as were serologies for HIV, HBV, and HCV. He had no diabetes (fasting glucose 97 mg/dL, HbA1c 5.4%). On ultrasound, kidney size was normal, and there were no signs of obstruction or lithiasis. 

On renal biopsy findings, 7 of 16 glomeruli were globally sclerotic; the remaining were hypertrophied with Bowman’s capsule thickening and hyaline expansion of the mesangial matrix, forming nodules (positive periodic acid Schiff stain), accompanied by slight segmental mesangial proliferation and mesangiolysis. Two glomeruli showed lesions of segmental sclerosis. There was severe interstitial fibrosis and tubular atrophy involving ~ 70% of the sample, accompanied by chronic inflammatory lymphocytic infiltrate. Arteries exhibited reduplication of the internal elastic lamina, and arterioles presented exuberant hyalinosis of the wall, with almost complete occlusion of the lumen (Congo red negative). The immunofluorescence study was negative. 

Ultrastructural evaluation: globally thickened basal membrane; podocytes with extensive foot process effacement. Mesangial matrix segmental expansion, with no increase of cellularity. No electron-dense deposits or fibrils were found. 

The patient never quit smoking, and despite blood pressure control with a maximum tolerated dose of perindopril, he progressed to stage G5A3 chronic renal disease within 3 years and was started on hemodialysis. 

## Non-diabetic metabolic nodular glomerulosclerosis 

### Definition 

NDMNG is a histologically based exclusion diagnosis, after ruling out: chronic membranoproliferative glomerulonephritis, dysproteinemia-related glomerular involvement, fibrillary or immunotactoid glomerulonephritis, fibronectin glomerulopathy, collagen III glomerulopathy, chronic hypoxic or ischemic conditions, and cystic fibrosis [3, 5, 6]. This pattern of glomerular lesion is a rare finding in the absence of diabetes, described in only 0.45 – 0.5% of biopsies [3, 7]. The so-far biggest meta-analysis described in the literature included only 95 cases [4]. 

Most recently, there has been discussion about the right nomenclature for this disease. After the established association between this histologic pattern with cardiovascular risk factors, the term “idiopathic nodular glomerulosclerosis” is no longer appropriate. “Diabetic nephropathy without diabetes”, “smoking-related glomerulopathy”, and “smoking-associated nodular glomerulosclerosis” are some of the proposed terminologies [4, 6, 8]. Since smoking is not the only predisposing identified risk factor for this condition, we consider that the term “non-diabetic metabolic nodular glomerulosclerosis” should instead be used. 

### Clinical presentation and outcomes 

NDMNG is more frequent among men, with a mean age of 60-years at biopsy (Table 1) [3, 4, 7, 8, 9, 10]. 

Patients usually present advanced kidney dysfunction, with serum creatinine ranging from 2.4 to 4.2 mg/dL. Urinalysis typically shows proteinuria and microhematuria ranges from absence to 75% in one series. The mean proteinuria at kidney biopsy is 1.9 – 4.2 g/day, and 22 – 53% may present with full nephrotic syndrome [3, 7, 8, 9, 10]. 

Studies have shown that these patients have a poor prognosis, with 30 – 35% reaching end-stage renal disease (ESRD) less than 3 years after biopsy [4]. On the other hand, smoking cessation and therapy with renin-angiotensin-aldosterone system inhibitors have been associated with better prognosis [3]. 

### Biopsy findings 

**Figure 1:**
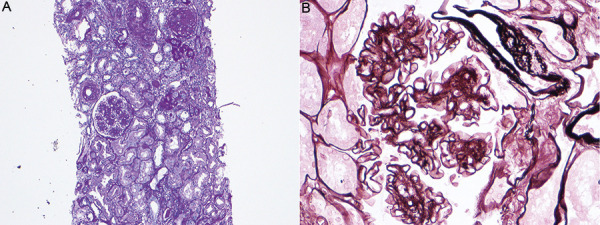
Renal biopsy. A: Periodic acid-Schiff stain (× 100) – 2 sclerotic glomeruli, 1 with nodular matrix expansion, arterioles with exuberant hyalinosis of the wall; B: Silver stain (× 400) – Matrix nodular expansion, slight dilation of the capillary walls (microaneurysms), a sign of mesangiolysis.

Nodular glomerulosclerosis presents histologically as an increase in the mesangial matrix, with nodule formation and glomerulomegaly, accompanied by thickening of the glomerular basal membrane, indistinguishable from diabetic nephropathy. More than 80% of patients show moderate to severe arteriolar hyalinosis and sclerosis [3, 4, 6]. Glomerular hyalinosis, mesangiolysis, and microaneurysms may be also observed (Figure 1). 

Tubulointerstitial fibrosis and tubular atrophy as well as arteriolar hyalinosis have been described as prognostic factors [3, 4]. 

Immunofluorescence is negative for immune deposits but can show IgG, IgM, C3, or albumin in an unspecific pattern [4, 6, 7, 9]. 

The ultrastructural evaluation shows thickened glomerular basement membrane (GBM) (> 400 nm in females, > 450 nm in males) and foot process effacement. Electron-dense deposits or fibrils are absent. The GBM is thickened, and there is podocytes foot process effacement (Table 2) [Table Table3] [3, 7, 9]. 

## Discussion 

NDMNG is a rare disease that has been associated with smoking and hypertension, among other cardiovascular risk factors. 

Hypertension is not only one of the most common etiologies of end-stage renal disease, but it is also present in more than 90% of NDMNG patients at the time of biopsy [3, 7, 8, 9, 10]. 

In patients with NDMNG, a high prevalence of smokers is noted – more than two-thirds of patients with a history of tobacco use and high cumulative intake of cigarettes, median 20 – 54 pack-years [3, 7, 9]. Smoking is a well-recognized risk factor for chronic kidney disease and even in non-diabetic and non-hypertensive patients, an association with microalbuminuria can be found [11]. The proposed mechanism by which smoking can lead to proteinuria and renal damage is through tobacco-sourced advanced glycation end product interaction with serum proteins [3, 6]. Cigarette smoking also produces free radicals that induce oxidative stress and activate the sympathetic nervous system, which induces activation of the renin-angiotensin-aldosterone system [3, 6, 7, 11]. Thus, smoking alters intrarenal hemodynamics, through vasoconstriction and blood flow reduction [6, 11]. All these mechanisms converge to produce a nephrotoxic effect that can be responsible for nodular glomerular lesions. 

NDMNG has been associated with several cardiovascular risk factors besides smoking and hypertension, such as obesity and hypercholesterolemia, which are also well-known vasculopathy promoters [3, 4, 5, 7, 8, 9]. We postulate that this lesion might arise in the presence of different combinations of cardiovascular risk factors and that it represents a severe form of metabolic microvasculopathy. 

The clinical case presented here is another example of the complex integration of risk factors, such as hypertension, smoking, and dyslipidemia, in the etiology of this lesion pattern, and consequent rapid progression to ESRD. More studies are needed to further improve knowledge about this entity and to clarify what is the best approach to prevent these patients from progressing to ESRD. 

## Funding 

This work has not received any contribution, grant or scholarship. 

## Conflict of interest 

The authors declare that there are no competing interests regarding the publication of this article. 

**Table 1.** Case series of non-diabetic metabolic nodular glomerulosclerosis – clinical characteristics.

	Markowitz et al. (2002) [3]	Li and Verani (2008) [9]	Wu et al. (2013) [7]	Salvatore et al. (2015) [8]	Hamrahian et al. (2018) [10]
Number of patients	23	15	20	4*	17
Male, %	78	33	80	100	76
Race					
White, %	74	73		100	41
Black, %	26	20		0	41
Asian, %	0	0		0	6
Hispanic, %	0	7		0	6
Media age, years (min – max)	68 (47 – 80)	64 (48 – 76)	56 (16 – 71)	62 (48 – 72)	60 (27 – 81)
Hypertension, %	96	93	90	100	100
Duration of hypertension, years	15	14	4		
Smokers, %	91	67	85	100	65
Cumulative cigarette intake, pack-years	53	54	20	23 years	
Obesity (BMI > 30), %	13	60	55		35
Overweight (BMI 25 – 29), %		27	40		35
Hypercholesterolemia, %	90		50		
Median creatinine at the diagnosis, mg/dL (min – max)	2.4	2.8 (1 – 6.8)	4.2 (1.1 – 8)	1.9 (1.3–3.0)	2.35
Median proteinuria, g/day (min – max)	4.7	5.6 (1 – 11.3)	2.85 (1.26 – 6.11)	3.0 (1.75 – 5.5)	3.58 g/g (0.8 – 12.3)
Nephrotic-range proteinuria, %	70	73	35		
Nephrotic syndrome, %	22	53			
Microhematuria, %			75	25	
ESRD, %	35		30	25	

*Six patients were excluded for nodular glomerulosclerosis pattern absence. ESRD = end-stage renal disease.

**Table 2.** Histologic findings in published case series.

	Markowitz et al. (2002) [3]	Li and Verani (2008) [9]	Wu et al. (2013) [7]	Salvatore et al. (2015) [8]*
Light microscopy				
Global glomerulosclerosis, %			85	24
Nodular mesangial matrix, %	100	100	100	100
Thickening of glomerular basement membrane, %	100	100	100	
Glomerulomegaly, %	100	100	100	100
Interstitial fibrosis and/or tubular atrophy, %	100	100	100	100
Mild (< 25%)	52	20	20	Mean 48 (35 – 60)
Moderate (25 – 50%)	61	53	30	
Severe (> 50%)	22	27	50	
Arteriosclerosis, %	100	100		100
Mild	17	0		0
Moderate	61	73		25
Severe	28	27		75
Hyalinosis and arteriolosclerosis, %	100	100	100	
Mild	4	0	20	
Moderate	57	53	70	
Severe	39	47	10	
Immunofluorescence				
Immune deposits / reactivity, %	0	0	0	0
Non-specific IgM/C3, %	70	80	Infrequent and unspecific patterns, IgG, IgM, C3, and albumin	
Linear staining of GBM and TBM for albumin and IgG, %	44 IgG, 48 albumin	53		
Electron microscopy				
Dense deposits, %	0	0	0	
Fibrils, %	0	0		
GBM thickening, %	100	100	100	100
Foot process effacement, %	46 (fusion)	100	100	100
Diffuse, %		67	65	
Segmental, %		33	35 (focal)	

*Six patients were excluded for the absence of nodular glomerulosclerosis pattern. GBM = glomerular basement membrane; TBM = tubular basement membrane.

**Table 3. Table3:** Non-diabetic metabolic nodular glomerulosclerosis summary.

Non-diabetic metabolic nodular glomerulosclerosis – key concepts	
Clinical presentation	Male > female Advanced age (+/– 60 years old) Late diagnosis (Advanced kidney dysfunction at diagnosis) Proteinuria (frequently nephrotic) +/- microhematuria
Comorbidities	Hypertension Smoking Obesity Hypercholesterolemia
Biopsy	Light microscopy: Nodular glomerulosclerosis, glomerulomegaly arteriosclerosis, hyalinosis, tubulointerstitial fibrosis Immunofluorescence: Negative/unspecific Electron microscopy: Thickening of GBM, podocytes foot process effacement no dense deposits or fibrils, thickening of GBM, podocytes foot process effacement
Treatment	Risk factors control (smoke cessation, hypertension control) Maximum tolerated dose of ACEi/ARA
Prognosis	Poor, 30% reach ESRD
